# Enhanced Production of Green Tide Algal Biomass through Additional Carbon Supply

**DOI:** 10.1371/journal.pone.0081164

**Published:** 2013-12-04

**Authors:** Pedro H. de Paula Silva, Nicholas A. Paul, Rocky de Nys, Leonardo Mata

**Affiliations:** School of Marine and Tropical Biology & Centre for Sustainable Tropical Fisheries and Aquaculture, James Cook University, Townsville, Australia; Mount Allison University, Canada

## Abstract

Intensive algal cultivation usually requires a high flux of dissolved inorganic carbon (Ci) to support productivity, particularly for high density algal cultures. Carbon dioxide (CO_2_) enrichment can be used to overcome Ci limitation and enhance productivity of algae in intensive culture, however, it is unclear whether algal species with the ability to utilise bicarbonate (HCO_3_^−^) as a carbon source for photosynthesis will benefit from CO_2_ enrichment. This study quantified the HCO_3_^−^ affinity of three green tide algal species, *Cladophora coelothrix*, *Cladophora patentiramea* and *Chaetomorpha linum,* targeted for biomass and bioenergy production. Subsequently, we quantified productivity and carbon, nitrogen and ash content in response to CO_2_ enrichment. All three species had similar high pH compensation points (9.7–9.9), and grew at similar rates up to pH 9, demonstrating HCO_3_^−^ utilization. Algal cultures enriched with CO_2_ as a carbon source had 30% more total Ci available, supplying twenty five times more CO_2_ than the control. This higher Ci significantly enhanced the productivity of *Cladophora coelothrix* (26%), *Chaetomorpha linum* (24%) and to a lesser extent for *Cladophora patentiramea* (11%), compared to controls. We demonstrated that supplying carbon as CO_2_ can enhance the productivity of targeted green tide algal species under intensive culture, despite their clear ability to utilise HCO_3_^−^.

## Introduction

Macroalgal biomass is an emerging resource for sustainable bioenergy [1] and advanced biofuels [2], [3]. Bioenergy applications rely on the production of a high volume/low value biomass opening opportunities to develop the culture of new commercial species. Green tide algae have the potential to meet these criteria as they are fast growing species [4] with a tolerance to a broad range of environmental conditions [5]. Furthermore, they are highly suitable as a bioenergy feedstock for ethanol [6], biogas [7], [8] and thermo-chemical conversion to biocrude [3], [9]. Green tide algae can be cultured extensively in open water culture [10] or harvested from natural blooms [11]. Alternatively, they can be cultured intensively in land-based ponds and tanks integrated into nutrient-rich aquaculture [12]–[14] and municipal [15] waste streams for bioremediation.

Dissolved inorganic carbon (Ci) is usually the limiting factor for growth in intensive cultivation with nutrient-rich systems, as the rate of Ci assimilation by the algae is greater than the rate of CO_2_ diffusion from the air into the water, even when vigorous aeration is used [16]. Total Ci in the water is composed of an equilibrium between carbon dioxide (CO_2_), bicarbonate (HCO_3_^−^) and carbonate (CO_3_^2−^), which are part of a buffered system. The relative amount of each fraction is dependent on pH, and to a lesser extent on salinity and temperature [17]. At pH 6, the molar fraction of the total Ci is divided equally between CO_2_ and HCO_3_^−^, the only usable forms of carbon for most of algae. The concentration of CO_2_ at pH 8.5 is negligible, as HCO_3_^−^ is in equilibrium with CO_3_^−2^, which is not a direct source of inorganic carbon for algal photosynthesis [18]. Above pH 9, the relative fraction of HCO_3_^−^ continues to decrease relative to CO_3_^−2^ leading to Ci limitation. Ironically, the daily pH fluctuations in a carbon limited system of intensive algal cultivation can cross at least 2 pH units [19], which provide a unique setting to evaluate the benefits of dosing Ci at commercial scales.

Increasing total dissolved CO_2_ concentrations in land-based intensive seaweed cultivation can therefore significantly enhance biomass productivity [20]–[23]. However, CO_2_ enrichment can also have no effect or may even be detrimental for some species [24]–[26]. The lack of widespread positive responses to CO_2_ enrichment in algae has been attributed to the presence of carbon concentration mechanisms (CCMs). These mechanisms allow algae to utilize the HCO_3_^−^ pool in seawater, which is the most common form of carbon [27]. However, the efficiency of HCO_3_^−^ use is species specific, with some species relying on HCO_3_^−^ to complement CO_2_ as a carbon source, while others can efficiently saturate carbon requirements using HCO_3_^−^ alone [28]. Therefore, enhanced productivity through CO_2_ enrichment is affected by the capability and efficiency with which species use HCO_3_^−^. Quantifying and understanding the response of algae to CO_2_ enrichment is a critical first step in optimisation of growth under intensive culture.

The major objective of this study was to quantify the ability of three green tide algal species, *Cladophora coelothrix* Kützing, *Cladophora patentiramea* (Montagne) Kützing and *Chaetomorpha linum* (O. F. Müller) Kützing, to utilise alternative carbon sources under intensive culture, and subsequently quantify their growth response to removing carbon limitations. These three species were selected as they are clearly identified targets for biomass production for bioremediation and bioenergy applications in tropical Australia [14], [29], and worldwide [8], [10], [30]. Specifically, we quantified the affinity for HCO_3_^−^ as a carbon source using the pH drift technique to determine the compensation point. We subsequently quantified growth under laboratory conditions at different pH levels with defined carbon sources. Finally, the three species were cultured for four weeks in an outdoor experiment testing the effects of CO_2_ enrichment and HCO_3_^−^ affinity on productivity and elemental composition (carbon, nitrogen and ash).

## Materials and Methods

### Algae collection and stock cultures

Three green tide algal species were collected from private aquaculture facilities in Queensland, Australia. *C. coelothrix* and *C. linum* were collected from the settlement pond and intake channel, respectively, of an intensive fish farm (Latitude: 20.02°S Longitude: 148.22°E, barramundi *Lates calcarifer*). *C. patentiramea* (Montagne) Kützing was collected from the intake dam of an intensive prawn farm (Latitude: 18.26°S Longitude 146.03°E, tiger prawns *Penaeus monodon*). Permission was obtained from owners to collect algae from these sites. Algal samples were hand collected and placed in aerated seawater for transportation to the James Cook University, Marine Aquaculture Research Facility Unit (MARFU). Stock cultures of each algal species were maintained in 70 L tanks within a recirculating system (∼27°C, 36‰).

### Algal affinity for HCO_3_^−^

Two approaches were used to quantify the ability of the three algal species to utilize HCO_3_^−^ as a source of Ci; pH drift technique (compensation point), and algal growth response to different pH levels.

### pH drift in closed vessel

The pH drift technique is a reliable method to determine HCO_3_^−^ utilization [31]. As the photosynthetic uptake of CO_2_ and/or HCO_3_^−^ results in a near stoichiometric production of hydroxyl ions, the pH of the culture media increases in response to photosynthesis. At pH 9, dissolved CO_2_ is virtually absent and species without mechanisms of HCO_3_^−^ utilization reach their limit of Ci extraction. Consequently, pH will not increase beyond this level, enabling the ability to utilise HCO_3_^−^ to be evaluated [32].

The pH drift assays were carried out in a culture chamber (Sanyo model MLR-351) with constant temperature (28°C) and irradiance (150 µmol photons m^−2^ s^−1^). Basal culture media was prepared using filtered sterile seawater (NO_3_-N 0.06 mg l^−1^, PO_4_^−^-P 0.02 mg l^−1^, Ci 1.9 mM and 32 ‰) enriched with f/2 growth media [33]. Algal samples were collected from the stock cultures, washed clean and pre-incubated for five days in the conditions described above. Approximately 100 mg fresh weight of filaments were incubated in closed airtight 120 ml graduated culture vessels filled with 130 ml of freshly prepared growth media (pH 7.9), leaving a minute air space. Culture vessels were repositioned and stirred hourly during the experiment to minimise any artefacts relating to light source or the formation of a boundary layer.

Thirty-six culture vessels were prepared for each species and three random culture vessels for each species (n = 3) were destructively sampled for pH measurements (YSI 63 pH meter). The pH drift assays ran for twelve hours. The pH measurements were performed at one and two hours in culture and then repeated every two hours until the maximum pH reached a stable level for at least two consecutive measurements (pH compensation point). This compensation point represents the pH at which the Ci taken up by the algae equals the CO_2_ released by respiration and/or photorespiration into the medium.

### Effects of pH on algal growth

The HCO_3_^−^ affinity of the algae can be inferred from their growth response at different pH levels because the relative amount of CO_2_ and HCO_3_^−^ available for growth is pH dependent. Above pH 8.5, where CO_2_ is virtually absent, species with no or little ability to use HCO_3_^−^ experience a steep decrease in growth. In contrast, species with the ability to efficiently use HCO_3_^−^ respond more slowly to the increase in pH as they utilize HCO_3_^−^ for growth.

To test HCO_3_^−^ affinity, algal biomass was transferred from the outdoor stock cultures to the laboratory and pre-cultured in f/2 enriched growth media for five days (in conditions described in the previous section). The growth experiment was carried out in a culture chamber (Sanyo model MLR-351) with constant temperature (28°C) and irradiance (150 µmol photons m^−2^ s^−1^) with a 12 L:12 D photoperiod. Samples of each algal species (∼100 mg fresh weight) were incubated in 100 mL of seawater enriched with f/2 growth media [33] within 120 mL plastic culture vessels with the lid loosely placed on top. The culture media in each treatment was buffered to maintain constant pH (+ 0.1 units), and correspondingly Ci ratios, using biological Tris (Sigma) at a final concentration of 25 mM. The water pH was adjusted to the desired pH levels (7, 7.5, 8, 8.5 and 9) using freshly prepared 1 M NaOH or HCl solutions. The culture media was prepared and replaced every day to renew Ci and to maintain the original CO_2_:HCO_3_^−^ ratios for each treatment. Algal filaments were filtered through a mesh screen and resuspended in the new growth media. Samples were again stirred and repositioned daily to a new position in the culture chamber. Treatments were weighted at the beginning and end of a ten day experimental period. Daily growth rates (DGR; % day^−1^) were then calculated using the following equation: 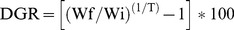
(1)where Wi is the initial fresh weight, Wf is the final fresh weight and T is the culture period in days.

### Algal productivity under CO_2_ enrichment

A CO_2_ enrichment experiment was performed outdoors using recirculating cultivation systems at the Marine Research Facility Unit (MARFU) at James Cook University between August and September 2010. Two independent sumps were used, one was supplied directly with CO_2_ gas stream (food grade 99.9% – BOC Australia) and regularly adjusted to maintain pH between 6.5 and 7, whereas the other acted as a control sump with no additional CO_2_. These systems provided a constant water flow of 2 volumes (vol) h^−1^ to polyethylene white buckets with 5 L capacity, 0.035 m^2^ surface area, containing a ring of aeration in the bottom to maintain the algae in tumble culture. The buckets were stocked with 3 g fresh weight L^−1^ (n = 3 for each species*CO_2_ treatment).

Cultures were acclimated for two weeks at these conditions and a formal growth experiment conducted over the subsequent four week period. Algal biomass of each tank was harvested weekly to determine productivity and subsequently restocked at the original density of 3 g fresh weight L^−1^. The algae were collected in mesh bags (0.1 mm mesh) and the biomass drained to a constant fresh weight in a washing machine (spin cycle 1000 rpm). Productivity (g m^−2^ day^−1^ dry weight) was then calculated using equation (2): 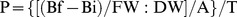
(2)where Bi is the initial biomass, Bf is the final biomass, FW:DW is the fresh to dry weight ratio, A is area of culture vessels and T the number of days in culture. The dry weights were acquired individually for each week from excess centrifuged biomass oven dried at 65°C for 48 h. Resulting FW:DW ratios were on average 3.5∶1 for *C. coelothrix*, 5∶1 for *C. patentiramea* and 5.9∶1 *C. linum*.

The water pH, temperature and salinity were measured daily in the inflow and outflow water of seaweed cultures at 08:00, 12:00 and 18:00 using an YSI 63 multi-parameter meter. Throughout the experiments water temperature and salinity averaged 28°C (2±SD) and 35‰ (1±SD), respectively. Ambient surface photosynthetic active radiation (PAR) was measured continuously using a LI-192S (2p) sensor placed near the tanks. Daily average PAR recorded during light hours for the experimental period was 881±152 µmol photons m^−2^ s^−1^. Water samples were collected twice a week at 12:00 from the inflow and outflow of tumble cultures for alkalinity determination. The samples were fixed with 200 µM of saturated HgCl_2_ solution, immediately taken to the lab and stored in the fridge until alkalinity analysis. Alkalinity was calculated using potentiometric titration by the Australian Centre for Tropical Freshwater Research (ACTFR) at James Cook University. Ci concentration and sources were calculated using the pH, alkalinity, salinity, and temperature original values of collection time, using the software CO_2_sys [34]. Nitrogen and phosphorus were measured from water samples collected from the inflow and immediately analysed by cadmium reduction and ascorbic acid techniques (HACH model DR/890), respectively. Average nitrogen and phosphorus concentrations during the experiment were ∼2.4 and 0.16 mg L^−1^, respectively.

### Biomass elemental analysis

The biomass of each tank was harvested at the completion of the four week growth period for elemental analysis (n = 3 for each CO_2_*species treatment). Biomass was spun dry and then oven dried at 60°C for 48 h, milled and stored in glass containers prior to analysis. Nitrogen and carbon were quantified for each sample using isotope analysis. Ash was quantified using a Carlo-Erba elemental autoanalyzer (Environmental Biology Group, Australian National University, Canberra).

### Statistical analysis

Two-way fixed-effect analyses of variance (ANOVA) were used to compare the growth response of the three green tide algal species to different pH and correspondent CO_2_ and HCO_3_^−^ ratios, and the effects of CO_2_ enrichment on biomass productivity using the software SYSTAT 12. Post-hoc comparisons were made to assess the differences between treatments in both experiments (Tukey's HSD multiple comparisons). The ANOVA assumptions of homogeneity of variance and normality were assessed by scatter plots and normal curve of the residuals, respectively [35]. To test whether the elemental composition of biomass was influenced by CO_2_ enrichment, we used a two-factor permutational multivariate analysis of variance (PERMANOVA, PRIMER v.6). The two fixed-factors were species and CO_2_ enrichment, while the dependent variables were the % nitrogen, carbon and ash content of dried biomass. Biomass composition data was fourth-root transformed for the PERMANOVA.

## Results

### pH drift in closed vessel

**Figure 1:**
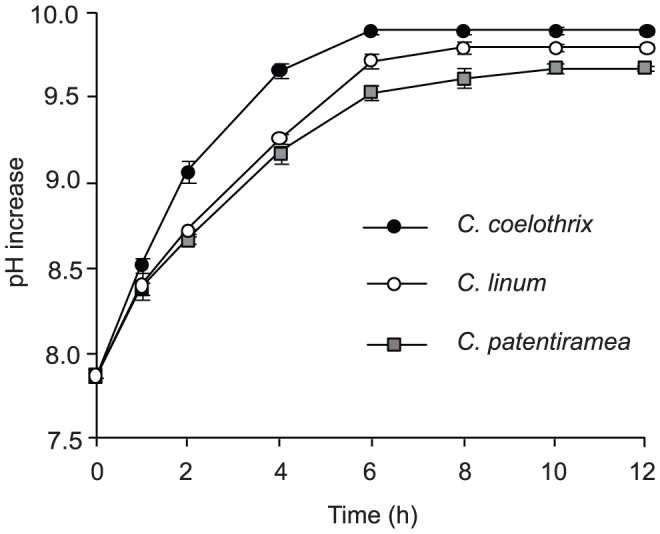
pH drift experiment for *C. coelothrix*, *C. patentiramea* and *C. linum.* Data show mean pH value (±1 SD) for each sampling time (n = 3).

The pH drifted from 7.9 to over 9.7 for all three algal species (Fig. 1). *C. coelothrix* had the highest pH compensation point of 9.9, which was reached after six h in culture. *C. linum* and *C. patentiramea* used the HCO_3_^−^ in the water at a slower rate, taking eight and ten hours to achieve the slightly lower pH compensation points of 9.8 and 9.7, respectively (Fig. 1).The relatively faster rate of pH increase for *C. coelothrix* supports more efficient HCO_3_^−^ use than either *C. linum* and *C. patentiramea*.

### Effects of pH on algal growth

**Figure 2:**
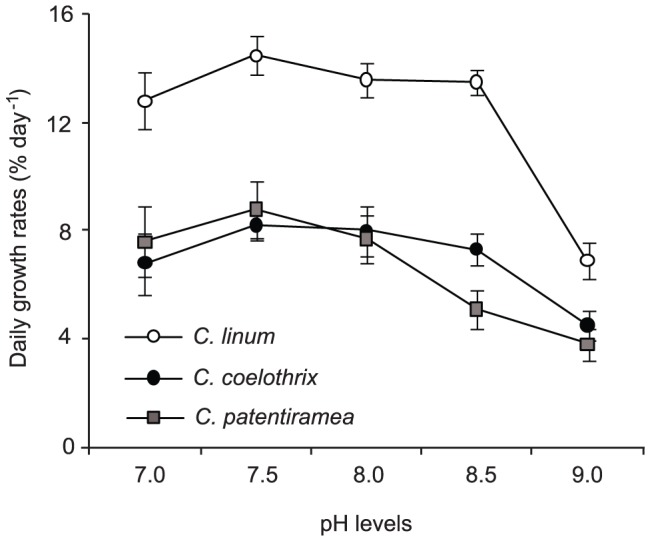
Growth of *C. coelothrix*, *C. patentiramea* and *C. linum* cultured in different pH levels. Data show mean daily growth rates (±1 SE) for each pH levels*species (n = 3).

All three species had decreasing growth rates with increasing pH above the optimum of pH 7.5. However, there was a significant interaction between the species and the pH levels in which they were cultured (P<0.001, Table 1, Fig. 2), driven by different optimal pH ranges for growth. In other terms, integrating the pH drift results in the previous section, different growth responses were reflective of different HCO_3_^−^ affinities. Both *C. coelothrix* and *C. linum* had higher growth rates at pH levels between 7 and 8.5, whereas the optimum pH range for *C. patentiramea* was between 7 and 8 (Fig. 2). There were no significant differences in growth rates within the optimal pH range for each species (Tukey's HSD, P>0.05). The highest individual growth rates for all three species were measured at pH 7.5, with growth rates of 14.5, 8.8 and 8.2% day^−1^ for *C. linum*, *C. patentiramea* and *C. coelothrix*, respectively (Fig. 2). Growth rates for *C. linum* and *C. coelothrix* decreased above the optimal pH range (from pH 8.5 to pH 9) by 48% and 35% relative to the control, respectively. Growth rates for *C. patentiramea* decreased by 30% relative to the control above the optimal pH range (from pH 8 to pH 8.5). Growth rate further decreased to 47% of the control at pH 9. The highest susceptibility of *C. patentiramea* growth to increasing pH levels (lower CO_2_:HCO_3_^−^ ratio) supports a relatively lower capability of using HCO_3_^−^. In accordance with the pH drift experiment, the lower sensitivity of *C. coelothrix* to changes in pH supports its more efficient use of HCO_3_^−^.

**Table 1:** Summary output for significant interactions of the ANOVA and PERMANOVA analyses.

Source	df	*MS*	*F*	*P*
**ANOVA**				
Species*pH	12	16.16	7.01	**<0.001**
Species* CO_2_	2	5.91	3.73	**0.031**
**PERMANOVA**				
Species* CO_2_	2	7.06	16.33	**<0.001**

ANOVA testing the effects of varying pH on growth and CO_2_ enrichment on algal productivity, and PERMANOVA (Species*CO_2_) testing effects of CO_2_ enrichment on biomass elemental composition.

### Algal productivity under CO_2_ enrichment

Based on the differences in HCO_3_^−^ utilization efficiencies between the three species, the subsequent step was to quantify increases in productivity through the addition of CO_2_ in controlled intensive cultures. The addition of CO_2_ decreased the pH of the inflowing seawater from ∼ pH 8 (control) to pH 6.7. Under these conditions the concentration of CO_2_ was twenty five times higher in the CO_2_ enriched cultures compared to the control (Table 2). The addition of CO_2_ also increased the concentration of HCO_3_^−^ in the CO_2_ enriched cultures to 2 mM, compared to 1.4 mM in the control cultures, because the hydration of CO_2_ produces carbonic acid, and its subsequent de-protonation leads to the formation of HCO_3_^−^. After passing through the seaweed tanks, at 2 vol h^−1^, all CO_2_ was depleted from water within the control cultures. In contrast, there was a continual supply of CO_2_ in the CO_2_ enriched cultures for photosynthesis. HCO_3_^−^ concentration in the control and CO_2_ enriched cultures was ∼1 mM and 1.5 mM, respectively. *C. coelothrix* cultures had the lowest concentration of all carbon forms (Table 2), and therefore the highest carbon uptake rates of all three species. *C. patentiramea* cultures had the highest Ci concentration in the water, in particular in the control treatment, and therefore carbon uptake rates were lower for *C. patentiramea* when only HCO_3_^−^ was present. These results confirm the laboratory data indicating that *C. patentiramea* has the least effective HCO_3_^−^ utilisation of the three species.

**Table 2:** Values for pH, dissolved inorganic carbon (Ci), carbon dioxide (CO_2_) and bicarbonate (HCO_3_^−^) for the CO_2_ enrichment experiments.

	CO_2_ enrichment	pH	Ci (mM)	CO_2_ (µM)	HCO_3_^−^ (µM)
Inflow	+CO_2_	6.73±0.26	2.33±0.20	250±60	1980±160
	Control	7.98±0.16	1.63±0.12	10±05	1400±100
*C. coelothrix*	+CO_2_	7.42±0.20	1.60±0.15	40±10	1510±140
	Control	8.52±0.12	1.17±0.13	0	930±130
*C. linum*	+CO_2_	7.38±0.19	1.62±0.14	50±10	1520±110
	Control	8.47±0.10	1.20±0.13	0	980±120
*C. patentiramea*	+CO_2_	7.35±0.20	1.62±0.11	50±10	1525±100
	Control	8.38±0.13	1.26±0.14	0	1100±140

Data show mean values (±1 SD) from the inflow and outflow of the green tide algal cultures with additional CO_2_ and control (n = 8).

**Figure 3:**
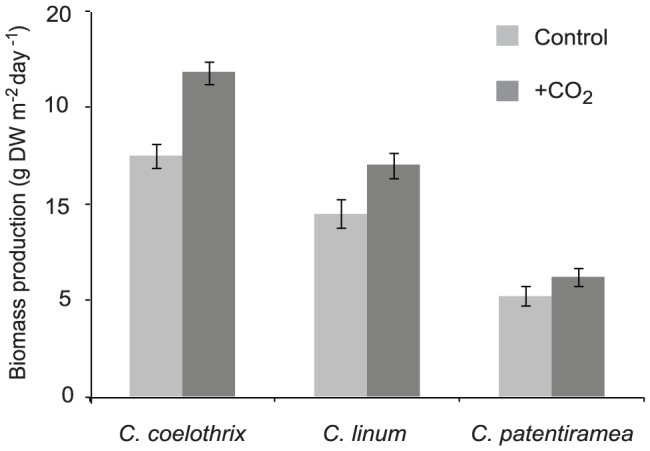
Biomass productivity in response to CO_2_ enrichment for *C. coelothrix*, *C. patentiramea* and *C. linum.* Data show mean biomass productivity (±1 SE) for each CO_2_ level*species (n = 3).

The three algal species had different productivity (growth) responses to CO_2_ enrichment, with a significant interaction between CO_2_ supply and the species tested (P<0.001, Table 1). The relative productivity of *C. coelothrix* and *C. linum* were significantly enhanced (∼26 and 24%, respectively) when supplied with additional CO_2_ (Fig. 3) The productivity of *C. coelothrix* increased from 12.5 to 16.8 g DW m^−2^ day^−1^, and *C. linum* from 9.5 to 12 g DW m^−2^ day^−1^ (Fig. 3). The productivity of *C. patentiramea* (5.2 to 6.2 g DW m^−2^ day^−1^) to CO_2_ enrichment was not significantly different to that of the control (Tukey's HSD, P>0.05).

### Biomass elemental analysis

In general, *C. coelothrix* and *C. linum* had higher carbon and nitrogen concentrations and lower ash contents than *C. patentiramea* (Table 3). However, CO_2_ enrichment influenced the elemental composition of the algal species in different ways (PERMANOVA, Species*CO_2_, P<0.001, Table 1). This interaction was mainly driven by positive influence of CO_2_ enrichment on carbon and nitrogen content in *C. coelothrix* and *C. linum* compared to the negative influence in *C. patentiramea*, which corresponded with an increase in ash content in the latter (Table 3). *C. coelothrix* biomass increased in carbon and nitrogen content by ∼2%, while ash decreased by ∼1% relative to the control (Table 3). *C. linum* biomass also increased in carbon and nitrogen content compared to the control, but to different degrees (by ∼4% and 2%, respectively). In contrast, the biomass of *C. patentiramea* cultured under CO_2_ enrichment had a lower and carbon (4%) and nitrogen (1%) content and high ash content (7%) compared with the control (Table 3).

**Table 3:** Values for % carbon (C), % nitrogen (N) and % ash from algal biomass cultured with CO_2_ enrichment and control.

Species	CO_2_ enrichment	% C	% N	% Ash
*C. coelothrix*	+CO_2_	33.29±0.28	6.05±0.03	25.20±0.55
	Control	30.97±0.30	4.07±0.03	26.22±0.56
*C. linum*	+CO_2_	31.67±0.70	5.98±0.10	29.20±0.88
	Control	27.12±0.13	4.12±0.13	33.43±0.47
*C. patentiramea*	+CO_2_	18.05±0.45	3.07±0.08	56.97±1.97
	Control	22.53±1.06	4.27±0.17	50.66±1.53

Data show mean values (±1 SD) of % dried biomass for each species* CO_2_ treatment (n = 3).

## Discussion

This study demonstrates that three green tide algal species, *C. coelothrix*, *C. linum* and *C. patentiramea*, have the ability to use HCO_3_^−^ as a complementary carbon source to CO_2_ for photosynthesis. However, this ability is restricted to a narrower pH range than for many other green algae belonging to the same genera. There is a lower comparative complexity or efficiency of the mechanisms involved in the uptake or conversion of HCO_3_^−^ to CO_2_ for these species. Consequently, this corresponds to a relatively higher dependence on CO_2_ as a carbon source for photosynthesis and this is reflected in the significant enhancement of productivity of these species when enriched with CO_2_ in intensive culture.

### Algal affinity for HCO_3_^−^pH drift in closed vessel

Comparatively, green algae as a taxonomic group photosynthesise at the highest pH levels with compensation points up to pH 10.8 [32]. At these pH levels, CO_2_ is absent and HCO_3_^−^ is the only functional form of inorganic carbon, representing less than a quarter of the total Ci. Active photosynthesis at these pH levels is only possible because of diverse and highly efficient mechanisms to overcome CO_2_ constraints through the utilization of HCO_3_^−^ [36]. There are at least two mechanisms to utilize HCO_3_^−^ in green macroalgae [37]. The first is the extracellular dehydration of HCO_3_^−^ into CO_2_ through the periplasmic carbonic anhydrase (CA) enzyme, followed by diffusion of CO_2_ into the cell, and this is the most widely distributed mechanism. The second mechanism is the direct uptake of HCO_3_^−^ through the plasma membrane, mediated by an anion exchange protein [38]. Some species of *Cladophora* have a third mechanism with the uptake the Ci through a vanadate-sensitive P-type H^+^-ATPase (proton pump) [39]. Species with these mechanisms raise the pH up to 10.5 in a closed vessel. The three species in this study did not raise the pH above 9.9 and therefore have limited HCO_3_^−^ transport. They almost certainly concentrate carbon using the dehydration of HCO_3_^−^ by CA into CO_2_, as this is the most common mechanism of HCO_3_^−^ utilization in algae [40]. This mechanism usually operates at ∼ pH 8.3, when the proportion of CO_2_ in the total Ci pool is below 1% and HCO_3_^−^ is more than 90%. The capacity to utilize HCO_3_^−^ through this mechanism decreases sharply with increased pH, and is ineffective at pH 9.8 [41]. The direct transport of HCO_3_^−^ through an anion exchange protein usually operates at higher pH (∼9.3) [39], and is the most probable mechanism for compensation points above 9.5 in the three species. These two mechanisms operate separately in other species of green algae with periplasmic CA activity dominating at lower pH, and direct uptake of HCO_3_^−^ by an anion exchanger at higher pH [39]. The incapacity of the species in this study to raise the pH above 9.7–9.9 suggests that there is no proton pump mechanism involved in HCO_3_^−^ transport. [42] inhibited the proton pump mechanism in *Ulva procera*, an alga capable of a pH compensation point of 10.5, and the pH remained below 9.9, demonstrating a reliance on this third mechanism to elevate the pH compensation to its highest level.

In a comparative context, the two *Cladophora* species in this study are less efficient in the use of HCO_3_^−^ than other species from the same genera with pH compensation points of ∼pH 10.5 [32], [42]. However, as in this study, some species of *Cladophora* maintain a preference for dissolved CO_2_ as a carbon source [43]. These different responses may be related to the environmental niche prior to experiments, as the ability of algae to utilise HCO_3_^−^ is strongly related to habitat [32]. Individuals of the same species can express alternate strategies for carbon acquisition when in different habitats, or the habitat itself might select for survival of genotypes with different carbon acquisition strategies [31]. This relatively limited ability to utilise HCO_3_^−^ is reflected in the growth response of all three species at different pH environments in this study.

### Effects of pH on algal growth

As pH increases from 7 to 8, the relative proportion of Ci present as CO_2_ is reduced by over 70%, while the relative proportion of HCO_3_^−^ decreases by only 10%. This drastic change in the CO_2_:HCO_3_^−^ ratio had no effect on the growth of algae in this study. The comparative ratio of CO_2_:HCO_3_^−^ was maintained at each pH throughout the experiment through the addition of a biological buffer. Consequently, CO_2_ is always available between pH 7 and 8 at concentrations that meet the carbon requirement for algal photosynthesis and growth. This does not, however, exclude the activation of the CA mechanism at ∼pH 8, which supplies additional CO_2_ derived from HCO_3_^−^ to compensate for its lower availability at increased pH [19].

From pH 8 to 8.5, CO_2_ decreases markedly and photosynthesis and growth depends on the efficiency of HCO_3_^−^ utilization mechanisms. The growth rates of *C. linum* and *C. coelothrix* within this pH range changed little. In contrast, a significant decrease in growth for *C. patentiramea* confirms that it is the least adapted to grow in the absence of CO_2_. Above pH 8.5, HCO_3_^−^ is replaced by CO_3_^2−^ and growth decreased significantly for all species. Steeper decreases in growth rates for *C. patentiramea* and *C. linum* between pH 8 and 9, compared to *C. coelothrix*, correspond with the slower rate of increasing pH for these two species in the pH drift experiment. These data, together with the highest pH compensation point, confirm that *C. coelothrix* has the most efficient mechanisms of HCO_3_^−^ utilisation. However, in a broader comparative context, the relatively low pH compensation points and significant decreases in growth rates from pH 8 to 9, again demonstrate that the three species in this study are not as efficient in the use of HCO_3_^−^ as a carbon source compared to many other green tide algal species.

### Algal productivity under CO_2_ enrichment

The productivity of two of the three species of green tide algae, *C. coelothrix* and *C. linum*, was enhanced through the addition of CO_2_. Notably, the enrichment treatment had twenty five times more CO_2_ available than the control. This maintained the pH of the enriched water below pH 7.5 (excess CO_2_), whereas the pH of the control cultures averaged 8.5 (depleted CO_2_). Considering the relatively limited ability of species to utilize the HCO_3_^−^ pool, and that this process has an energetic cost [28], the constant presence of CO_2_ at pH 7.5 disproportionately facilitated photosynthetic carbon fixation, and ultimately enhanced biomass productivity. Enhanced growth rates with CO_2_ enrichment have also been reported for other species capable of using bicarbonate [20]–[23]. However, the magnitude of the growth responses to CO_2_ enrichment is to some extent dependent on the efficiency of carbon concentrating mechanisms for each species. For example, species depending almost exclusively on CO_2_ for photosynthesis can increase their biomass productivity up to three times when cultured in enriched CO_2_ culture media [19], [44], [45]. Any differences in growth relative to enhanced CO_2_ can also be due to the effect of CO_2_ on the rate of nitrogen assimilation [46], [47]. High levels of CO_2_ can increase the rate of nitrogen assimilation in some algae by up-regulating nitrate reductase, the main enzyme in the nitrate assimilatory pathway [47], [48]. This may be the case for *C. coelothrix* and *C. linum* in this study where they have a higher nitrogen content under CO_2_ enrichment. Higher nitrogen and carbon contents on top of increased productivities with CO_2_ enrichment represents a clear advantage for integrated systems focused on biomass production for bioremediation of waste streams [14], [49].

In contrast, the nitrogen content of *C. patentiramea* decreased under CO_2_ enrichment, suggesting no effect on assimilation. The effect of high CO_2_ on the assimilation of nitrogen in algae is not consistent with decreases in assimilation for other species of algae [24], [50]. This effect may contribute to the relatively lack of increase in productivity of *C. patentiramea* under CO_2_ enrichment. Notably, laboratory experiments suggested that *C. patentiramea* should be the most sensitive species to CO_2_ enrichment based on relative capabilities of HCO_3_^−^ utilization, which indicates that controlled, static, laboratory experiments may not be efficient to predict responses in flow environments (e.g. similar to commercial scale), potentially because of boundary layer/water motion effects on Ci distribution [51]. An alternative but related driver to water motion is the morphological differences between the green tide algae. The two rapidly growing species which had enhanced growth under CO_2_ enrichment, *C. coelothrix* and *C. linum*, have a fine filamentous morphology suitable for tumble culture. In contrast, *C. patentiramea* has tightly interwoven filaments (e.g. ball-like structure) that restrict light to the inner filaments (auto-shading), thereby potentially limiting photosynthesis and growth. These physical factors may have influenced small density cultures in the laboratory in a different way than the dense cultures in the outdoor experiment, where individual, larger clumps could become limited. Regardless, *C. patentiramea* is not a good option for intensive cultivation because despite the lack of growth response to additional CO_2_, high CO_2_ affected negatively the nitrogen and carbon content while increasing ash content, and therefore the amount of biomass that can be converted into soil conditioners [52] or biofuels [9] decreases substantially.

In conclusion, intensive cultures of *C. coelothrix* and *C. linum* enriched with CO_2_ had significantly enhanced productivity, despite their ability to utilise HCO_3_^−^. This demonstrates the potential for enhanced production for these species using CO_2_ enrichment. This can be integrated with the industrial production of CO_2_ and waste water streams from industry [53] to deliver a model where algae provide a bioremediation service of both air (CO_2_) and water (nitrogen, phosphorous, metals and trace elements), and an opportunity to utilise this biomass for bio-energy products.
